# Trajectories of child cognitive development during ages 0–3 in rural Western China: prevalence, risk factors and links to preschool-age cognition

**DOI:** 10.1186/s12887-021-02650-y

**Published:** 2021-04-26

**Authors:** Lei Wang, Yifei Chen, Sean Sylvia, Sarah-Eve Dill, Scott Rozelle

**Affiliations:** 1 International Business School, Shaanxi Normal University, No. 620, West Chang’an Avenue, Chang’an District, Xi’an, 710119 Shaanxi China; 2 Department of Health Policy and Management, Gillings School of Global Health, Carolina Population Center, University of North Carolina at Chapel Hill, Chapel Hill, North Carolina USA; 3 Freeman Spogli Institute for International Studies, Stanford University, Stanford, California USA

**Keywords:** Cognitive development, Developmental trajectories, Early childhood, Rural Western China

## Abstract

**Background:**

Cognitive development after age three tends to be stable and can therefore predict cognitive skills in later childhood. However, there is evidence that cognitive development is less stable before age three. In rural China, research has found large shares of children under age three are developmentally delayed, yet little is known about the trajectories of cognitive development between 0 and 3 years of age or how developmental trajectories predict later cognitive skills. This study seeks to describe the trajectories of child cognitive development between the ages of 0–3 years and examine how different trajectories predict cognitive development at preschool age.

**Methods:**

We collected three waves of longitudinal panel data from 1245 children in rural Western China. Child cognitive development was measured by the Bayley Scales of Infant Development when the child was 6–12 months and 22–30 months, and by the Wechsler Preschool and Primary Scale of Intelligence-Fourth Edition when the child was 49–65 months. We used the two measures of cognitive development before age three to determine the trajectories of child cognitive development.

**Results:**

Of the children, 39% were never cognitively delayed; 13% were persistently delayed; 7% experienced improving cognitive development; and 41% experienced deteriorating development before age 3. Compared to children who had never experienced cognitive delay, children with persistent cognitive delay and those with deteriorating development before age 3 had significantly lower cognitive scores at preschool age. Children with improving development before age 3 showed similar levels of cognition at preschool age as children who had never experienced cognitive delay.

**Conclusions:**

Large shares of children under age 3 in rural Western China show deteriorating cognitive development from infancy to toddlerhood, which predict lower levels of cognition at preschool age. Policymakers should invest in improving cognitive development before age 3 to prevent long-term poor cognition among China’s rural children.

**Supplementary Information:**

The online version contains supplementary material available at 10.1186/s12887-021-02650-y.

## Background

Early childhood has been identified as an important window for cognitive development [1–5]. It is well known that cognitive development during the first 3 years of life is predictive of cognitive development in later childhood [6–9]. Theoretical and empirical research has established that basic cognitive skills developed in the earliest years of life form the foundation for the development of more complex skills later in childhood [4, 10, 11]. For this reason, infants who have delayed development during the first 3 years of childhood face impediments to developing more complex cognitive skills and are likely to continue to have low levels of cognitive skill into their preschool years [6, 7, 12–15]. Several studies have found that cognitive skills at age 3 were predictive of cognitive skills at preschool age (4 to 5) with coefficients of 0.36 to 0.64 [6, 8, 16].

Although the literature has consistently found that levels of cognition are relatively constant after a child is 3 years old [17, 18], a number of studies, especially in developed countries, have found that fluctuations in cognitive development before age 3 are relatively common [6, 19–24]. For example, a study by Feinberg et al. [21] found that 28% of cognitively delayed children in the United States were persistently delayed from 9 to 24 months old, while the remaining children who were delayed at 24 months (72%) were newly delayed (i.e., their levels of measured cognition had deteriorated between 9 months and 24 months). In another study from the United States, nearly 70% of sampled children with measured cognitive delay at 9 months had recovered to normal cognition by 24 months [23], while the remaining 30% of children remained delayed at 24 months [23]. Although the changes in cognitive development over time may be due to measurement error, the measurements used in these studies have high reliability, and most of the changes are likely to represent true fluctuations in cognitive development.

A small number of studies have provided evidence that trajectories of cognitive development before 3 years of age affect later development, such as behavioral outcomes, at preschool age. To the best of our knowledge, such studies have been conducted only in developed countries [19, 23]. One study conducted in the United States, for example, demonstrated that different cognitive trajectories before 3 years were linked to differences in preschool-aged developmental outcomes [19]. Compared to children who never experienced cognitive delay (at age 5), persistently delayed, improving, and deteriorating children were shown to have higher frequencies of behavioral problems by 0.6 standard deviations (SD), 0.2 SD, and 0.4 SD, respectively. Unfortunately, however, the study did not examine whether the trajectories of cognitive development in early childhood were correlated with cognitive outcomes at preschool age.

Understanding the trajectories of cognitive delay and their relation to later cognitive skills should be especially relevant for developing countries, where research has shown that about 250 million children under the age of 5 (about 43%) are at risk of developmental delay [25]. Of this global total, it is estimated that 45 million are in China, which would make China rank second globally in terms of total number of young children with cognitive delay [25–27]. In China, recent studies suggest that cognitive delay is most prevalent among children in rural areas. Whereas research in urban areas has consistently shown rates of cognitive delay among infants and toddlers of under 15%, the average rate of delay for a healthy population [28–30], studies of children aged 0 to 3 years in rural China have found rates of cognitive delay between 39 and 49% [26, 31, 32]. Although fewer recent studies have examined the cognitive development of preschool-age children in rural China, the existing studies have found that the rates of cognitive delay of preschool-age children are similarly high: a 2008 study of 505 low-income, rural children aged 4 to 5 found a rate of cognitive delay around 57% [33], and a more recent study conducted in Guizhou province in 2015 found that over half of rural children aged 64–71 months were cognitively delayed [34].

Although previous studies in rural China have not focused on the trajectories of cognitive skills, there is evidence that suggests that cognitive development children under age 3 in rural China may fluctuate over time. A longitudinal study of young rural children in China found the rate of cognitive delay of sample children increased from 14% at 6 months to 49% at 29 months [35]. Another study, which examined the impacts of a parental training intervention to increase psychosocial stimulation, found that it was possible to improve the cognitive development of the sample children, especially those children who had low levels of cognition at baseline [36]. Importantly, however, no study in China to date has documented the trajectories cognitive development among young children under age 3. Additionally, little is known about how different trajectories of cognitive development before age 3 may predict cognitive development as children grow older (e.g., to preschool age).

If the trajectories of cognitive development during 0 to 3 years of age are associated with development in later childhood (both preschool age and beyond), there would be great value for researchers and policymakers to determine the factors linked with the different trajectories. To our knowledge, however, only one study has examined factors associated with the different trajectories of cognitive development among young children [19]. This study, conducted by Cheng et al. (2014) among children in the United States, found that trajectories of cognitive development from infancy to preschool age were related to the demographic characteristics of children and families: children with low birth weight and those from families with low income were more likely to experience persistent cognitive delay, whereas female children and those with siblings were more likely to see their cognitive development improve. Additionally, although no studies in developing countries have examined cognitive trajectories before age 3, many studies in developing counties have identified risk factors that are correlated with early childhood cognitive delay, including low parental education levels, low family income, and greater number of siblings in a family [26, 35, 37, 38]. Given the consistency of these findings across settings as diverse as Columbia, China, and South Africa, it is possible these factors also may be related to trajectories of child cognitive development.

The aim of this paper is to describe the trajectories of child cognitive development between the ages of 0–3 years among in rural Western China and examine how different trajectories predict cognitive development at preschool age. To achieve this goal, we have four specific objectives. First, we describe child cognitive development at three points in time: infancy (6–12 months), toddlerhood (22–30 months) and preschool age (49–65 months). Second, we describe the trajectories of child cognitive development from infancy to toddlerhood and report the shares of sample children who are never delayed, persistently delayed, show improving cognitive development and show deteriorating cognitive development before age 3. Third, we examine how the different trajectories of cognitive development before age 3 predict cognitive skills at preschool age. Finally, we identify individual and household factors that are associated with each developmental trajectory before age 3, including the child’s age, gender, whether the child was born prematurely, whether the child had siblings, maternal age, maternal education level, and the family asset index of each household.

The remainder of this paper is structured as follows. Section 2 presents our methods, including sample selection, data collection, and statistical methods. Section 3 describes the results. Section 4 discusses the findings, and section 5 concludes.

## Methods

### Sample selection

The data presented in this paper come from a longitudinal study of children and households conducted by the authors in 11 nationally designated poverty counties^1^ in the Qinba Mountain area of China. This region is nationally recognized as a concentrated and contiguous poverty-stricken area in China [39]. In 2013, the per capita GDP of the region was US$1275 (RMB 7896), lower than the national per capita GDP of US$7057 (RMB 43,684) [40]. Of the 75 counties in the region, nearly all are designated as poverty counties by the central government of China [39].

The sample was selected in 2013 using a multistage cluster sampling design. First, all townships in the 11 counties were included in the study, with two exceptions: We excluded the one township in each county that housed the county seat and any townships that did not have any villages with a population of 800 or more. In total, according to these criteria, 174 townships were included in the study. Next, we randomly selected two villages from each of the sample townships. Finally, with the help of the local family planning offices in the study area, we obtained a list of all registered children born between March 2012 and May 2013 in each sample village. We excluded infants with known diseases or disabilities and selected all remaining infants within the target age range (6–12 months) for inclusion in this study. Overall, the baseline sample included 1802 children.

Following the initial survey wave in 2013, we conducted two follow-up surveys: one in April 2015, when the sample children were 22–30 months old, and another in 2017, when the sample children were 49–65 months old. We use child cognitive development measured in the first and second survey waves to determine trajectories of development before age 3, while cognitive skills assessed during the third survey wave serve as our main outcome variable. In total, 1272 children were surveyed in all three waves. It should also be noted that there were missing data from children who did not complete the Bayley Scales of Infant Development (BSID) and the Wechsler Preschool and Primary Scale of Intelligence-Fourth Edition (WPPSI-IV) (4 in the first wave, 10 in the second wave, and 13 in the last wave). We performed a series t-tests to compare those children not having cognitive scores at any wave with those having cognitive scores at all three waves (Additional file 1: Appendix Table A1). No significant differences were detected on any demographic variable. These data give evidence that excluding the missing values probably did not bias the results. For our analysis, we excluded the sample not having cognitive scores at any wave. Our final sample included 1245 children.

### Data collection and measures

In each survey wave, we collected two main blocks of data. The first block collected data on the cognitive development of each sample child. The second block collected data on the demographic characteristics of sample children and households.

#### Cognitive development

Because child cognitive assessments are typically designed for specific age ranges, we use two different assessments of cognition based on the ages of the sample. During the first and second survey waves (when children were 6–12 months and 22–30 months, respectively), we assessed cognitive development using the first version of the Bayley Scales of Infant Development (BSID). The BSID is an internationally-recognized scaled test for children under 30 months [41–43]. This test was formally adapted to the Chinese language and culture in 1993 and scaled according to an urban Chinese sample [44]. The BSID produces a mental development index (MDI) that measures memory, habitation, problem solving, early number concepts, generalization, classification, vocalizations, and language [45]. In the Chinese version of the BSID, the MDI has an inter-rater reliability of 0.99, a test-retest reliability rate of 0.82, and a parallel forms reliability of 0.85 [46]. The MDI has an expected mean of 100 and SD of 16. Children with an MDI score below 84 (1 SD) are considered developmentally delayed.

In the third survey wave (when sample children 49–65 months), we assessed cognitive development using the Chinese version of the Wechsler Preschool and Primary Scale of Intelligence-Fourth Edition (WPPSI-IV). The WPPSI-IV is an individually-administered, standardized test for assessing the cognitive functioning of children aged 30–91 months [47]. The Chinese version of the WPPSI-IV was adapted in 2010 and scaled according to a Chinese sample from urban and rural areas [48], and has since been applied in research across China [49, 50]. The WPPSI-IV produces a Full-Scale Intelligence Quotient (FSIQ), which is a composite score that summarizes cognitive ability across a diverse set of domains. The Chinese version of the WPPSI-IV test has a reliability coefficient of 0.96 for the FSIQ. Considering the Flynn effect calculated based on Flynn, Trahan et al. and Wang et al. [51–53], children with FSIQ scores more than 1 SD lower than the mean of 101.3 are considered developmentally delayed.

The BSID and the WPPSI-IV were administered one-on-one to each child by trained testers, using a standardized set of toys and a detailed scoring sheet. The testers underwent a formal week-long training course, including 2.5 days of field training, prior to each survey wave. All of the BSID assessments were conducted in the home for each child. All of the WPPSI-IV assessments were conducted at either the child’s home or preschool. Neither caregivers nor teachers were allowed to assist the child during the administration of the tests.

#### Demographic characteristics

Teams of trained enumerators collected child and household characteristics from each sample child’s primary caregiver (defined as the individual most responsible for the child’s daily care, typically the child’s mother or paternal grandmother). Child characteristics included the child’s age in months, gender, whether the child was premature (born before 37 weeks of gestation), and whether the child had siblings. Household characteristics included maternal age, maternal education level, and the family asset index of each household.^2^ Each child’s age and premature birth status were obtained from his or her birth certificate.

### Statistical analysis

For our analysis, BSID and WPPSI-IV cognitive raw scores are standardized separately for each survey wave. Because raw scores increase with age, we computed age-adjusted standardized cognitive scores by subtracting age-specific means and dividing by age-specific SDs, estimated using non-parametric regression methods. This method is used mainly because the number of sample observations in each age segment is relatively small, and this procedure makes the data less sensitive to outliers [54]. Using this approach yields normally distributed standardized scores with a mean of zero across the age range.

Following Cheng et al. and Witt et al. [19, 24], we sort sample children into four groups based on their trajectory of cognitive development from infancy (6–12 months) to toddlerhood (22–30 months). These four categories are: 1) “never” cognitively delayed, defined as having no cognitive delay at either 6–12 months or 22–30 months; 2) “persistently” cognitively delayed, defined as having cognitive delay at both 6–12 months and 22–30 months; 3). “improving,” defined as having cognitive delay at 6–12 months but no longer delayed by 22–30 months; or 4) “deteriorating,” defined as not having delayed at 6–12 months but developing cognitive delay at 22–30 months.

To examine associations between trajectories of cognitive development before age 3 and cognitive skills at preschool age (49–65 months), we employed ordinary least squares (OLS) to construct a model as follows:

1 $$ {Cognition\ Outcomes}_i={\beta}_0+{\beta}_1{Evolutionary\ Path}_i+{X}_i+{u}_i, $$

where *Cognition Outcomes*_*i*_ represents the standardized FSIQ score of child *i* at preschool age (49–65 months). *Evolutionary Path*_*i*_ is a dummy variable for the trajectory of cognitive development of child *i* that is equal to 1 when a child is in the trajectory of interest and 0 otherwise. For example, when we examine the association of never having cognitive delay category before age 3 to FSIQ at preschool age, *Evolutionary Path*_*i*_ is equal to 1 for “never” and 0 otherwise. We compare all other trajectories in this way, using the *Evolutionary Path*_*i*_ variable in the same manner. The term *X*_*i*_ is a vector of covariates that are included to capture the individual and household characteristics of each child (age, gender, whether the child was premature, whether the child has siblings, MDI score at baseline, identity of primary caregiver, maternal age, maternal educational level, and family asset index). *u*_*i*_ is an error term. We also control for county fixed effects and time fixed effects.

We also used the same OLS regression approach with an alternative specification to estimate the associations between the four trajectories of cognitive development before age 3 and cognitive skills at preschool age. The model is constructed as follows:

2 $$ {Cognition\ Outcomes}_i={\beta}_0+{\beta}_1{Evolutionary\ Path}_i+{X}_i+{u}_i, $$

where the dependent variable, *Cognition Outcomes*_*i*_ represents the standardized FSIQ score of child *i* at preschool age (49–65 months). The variable, *Evolutionary Path*_*i*_, is a vector of three dummy variables that measure whether the child was in one of three trajectories: persistently delayed, improving, or deteriorating. The never delayed group is used as a reference group against which the other three groups are measured. As in Eq. (1), the variable *X*_*i*_ is a vector of covariates capturing child and household characteristics, and *u*_*i*_ is an error term. We also control for county fixed effects and time fixed effects.

To identify which of the child or household characteristics are most highly associated with each trajectory of cognitive development, we followed Cheng et al. (2014) and Witt et al. (2009) to construct a multivariate probit regression model (using a limited dependent which takes on a value of either 1 or 0) [19, 24]. The model is as follows:

3 $$ {Evolutionary\ path}_i={\beta}_0+{\beta}_1{\mathrm{Child}}_i+{\beta}_2{Household}_i+{u}_i, $$

When we compare the differences in characteristics between children who never experienced cognitive delay and children classified as deteriorating, the dependent variable, *Evolutionary path*_*i*_, equals 1 for “deteriorating” and 0 for “never.” When we compare the differences in characteristics between persistently cognitively delayed children and children who were improving, the dependent variable, *Evolutionary path*_*i*_, equals 1 for “improving” and 0 for “persistently.” Child_*i*_ represents individual child characteristics, including age, gender, whether the child was premature, and whether the child has siblings. *Household*_*i*_ represents household characteristics, including identity of the primary caregiver, maternal age, maternal educational level, and family asset index. *u*_*i*_ is a mean-zero error component, which captures unobserved factors that determine the dependent variable. We also control for county fixed effects and time fixed effects. To provide consistency with the models in Eqs. (1), (2) and (3), we also used OLS to estimate the model specified in Eq. (3) and have put the results in Additional file 1: Appendix Table A2.

Finally, we employed the same ordinary least squares (OLS) estimation approach as used to estimate Eqs. (1) and (2) to estimate the associations between preschool-age cognitive skills and child cognitive development outcomes at different age ranges. We use the following model:

4 $$ {Cognition\ Outcomes}_{i(preschool)}={\beta}_0+{\beta}_1{\mathrm{Cognition}\ \mathrm{Outcomes}}_{i(j)}+{X}_i+{u}_i, $$

where *Cognitive Score*_*i*(*preschool*)_ represents the standardized FSIQ score of child *i* at preschool age (49–65 months). When we evaluate the association between cognition outcomes at preschool age and in infancy (6–12 months), the independent variable, Cognitive Score_*i*(*j*)_, represents the standardized MDI score of child *i* in infancy. When we examine the association between cognition outcomes at preschool age and in toddlerhood (22–30 months), the independent variable, Cognitive Score_*i*(*j*)_, represents the standardized MDI score of child *i* in toddlerhood. When we assess the association between cognition outcomes at preschool age and changes in cognition scores from infancy to toddlerhood, Cognitive Score_*i*(*j*)_ represents the change in the standardized MDI score of child *i* from infancy to toddlerhood. The term *X*_*i*_ is a vector of covariates, as defined above in Eq. (1), and *u*_*i*_ is an error term. We control for county fixed effects and time fixed effects.

## Results

### Descriptive characteristics of participants

Table 1 presents the demographic characteristics of the sample children and their caregivers. Among the children in our sample, slightly more than half (51%) were male, and 76% did not have siblings. Only a small percentage of children (5%) were premature. For 85% of the sample children, the mother was the primary caregiver. In the case of the remaining 15% of the caregivers, the paternal grandmother was most often the primary caregiver. Among the mothers in our sample, 62% were more than 25 years old. Only 15% of the mothers had completed more than 12 years of schooling.

**Table 1:** Characteristics of sample children (6–12 months) (*N* = 1245)

Characteristic	Frequency (*n*)	Percentage (*%*) or Mean ± SD
Child		
Gender		
Male	640	51.4
Female	605	48.6
Whether the child was premature		
Yes	58	4.7
No	1187	95.3
Whether the child has siblings		
Yes	298	23.9
No	947	76.1
Household		
Mother is primary caregiver		
Yes	1058	85.0
No	187	15.0
Maternal age		
< 25	476	38.2
≥ 25	769	61.8
Maternal education level (years)		
< 12	1057	84.9
≥ 12	188	15.1
Family asset index	1245	−0.1 ± 1.2

Asset index constructed using polychoric principal components of the following variables: tap water, toilet, water heater, washing machine, computer, Internet, refrigerator, air conditioning, motorcycle or electronic bicycle, and automobile

### Child cognitive outcomes

For all survey waves (6–12 months, 22–30 months and 49–65 months), the mean cognitive scores of the sample children were lower than the mean of 100 found in healthy populations (Table 2). Similarly, the data show that a considerably large share of the sample children were suffering from cognitive delay (developmental scores 1SD or more below the healthy mean), with the rate of delay increasing significantly from 20% during infancy (6–12 months) to 55% by toddlerhood (22–30 months). When the sample children reached preschool age (49–65 months), the rate of cognitive delay remained high (45%). For all three survey waves, the rates of cognitive delay were higher than the rate found in a healthy population (15%) [53].

**Table 2:** Cognitive outcomes of rural young children in different age ranges in Northwest China (*N* = 1245)

Outcome	Infancy	Toddlerhood	Preschool Age	Diff. (1)–(2)	Diff. (1)–(3)	Diff. (2)–(3)
	Mean (SD)	Mean (SD)	Mean (SD)	*p*-value	*p*-value	*p*-value
Cognitive score	96.3 (16.70)	81.0 (21.49)	88.7 (11.75)	< 0.01	< 0.01	< 0.01
Rate of delay	20% (0.40)	55% (0.50)	45% (0.50)	< 0.01	< 0.01	< 0.01

Data source is author’s survey. Cognitive scores are the Bayley Scale of Infant Development (*BSID*) scores on the Mental Development Index (*MDI*) for infants (6–12 months) and toddlers (22–30 months), and the Wechsler Preschool and Primary Scale of Intelligence-Fourth Edition (*WPPSI-IV*) scores on the Full-Scale Intelligence Quotient (*FSIQ*) for preschool-age children (49–65 months). Delay is defined as having cognitive scores below − 1 standard deviation (*SD*) of the mean

### Trajectories of child cognitive development

Table 3 presents the shares of sample children in each of the four trajectories of cognitive development. Of the 1245 children in the full sample, 481 (39%) were never cognitively delayed; 165 children (13%) were persistently cognitively delayed; 83 children (7%) showed improving cognitive development; and 516 children (41%) experienced deteriorating cognitive development.

**Table 3:** Trajectory of child cognitive development from infancy to toddlerhood (*N* = 1245)

Development	Infancy	Toddlerhood	Frequency (*n*)	Percentage (*%*)
Never delayed	No	No	481	38.6
Persistently delayed	Yes	Yes	165	13.3
Improving	Yes	No	83	6.7
Deteriorating	No	Yes	516	41.4

“Never delayed” includes all young children whose scores on the Bayley Scale of Infant Development (*BSID*) of the Mental Development (*MDI*) in infancy and toddlerhood never fell below − 1 standard deviation (*SD*). “Persistently delayed” includes all young children whose scores on the MDI scales in infancy and toddlerhood never rose above − 1 SD. “Improving” includes all young children whose scores on the MDI scales fell below − 1 SD in infancy and then rose above − 1 SD in toddlerhood. “Deteriorating” includes all young children whose scores on the MDI scales were above − 1 SD in infancy and then fell below − 1 SD in toddlerhood. Data source is authors’ survey

### Associations between trajectories of cognitive development and cognitive outcomes

Table 4 compares the preschool-age FSIQ scores of sample children in each cognitive trajectory (never delayed, persistently delayed, improving, deteriorating) to the children in all of the other groups combined using Eq. (1). The results show that children who were never cognitively delayed before age 3 had significantly higher preschool-age FSIQ scores relative to children in the other three trajectories. Children with improving cognitive development also scored higher than children in the rest of the sample. In contrast, children with persistent cognitive delay and those whose cognitive development had deteriorated had significantly lower preschool FSIQ scores as compared to the rest of the sample.

**Table 4:** Ordinary Least Squares estimates of the association between cognitive score at preschool age and trajectory of child cognitive development from infancy to toddlerhood (*N* = 1245)

Development	Standardized FSIQ scores (at preschool age)			
	(1)	(2)	(3)	(4)
Never delayed (1 = never, 0 = otherwise)	0.50*** (0.06)			
Persistently delayed (1 = persistently, 0 = otherwise)		−0.53*** (0.09)		
Improving (1 = improving, 0 = otherwise)			0.55*** (0.11)	
Deteriorating (1 = deteriorating, 0 = otherwise)				−0.41*** (0.05)
Control variables	Yes	Yes	Yes	Yes
County fixed effects	Yes	Yes	Yes	Yes
Time fixed effects	Yes	Yes	Yes	Yes
*R*-squared	0.22	0.20	0.19	0.21

Models estimated in this table are defined in Eq. (1) in the manuscript. “Never delayed” includes all young children whose scores on the Bayley Scale of Infant Development (*BSID*) of the Mental Development (*MDI*) in infancy and toddlerhood never fell below − 1 standard deviation (*SD*). “Persistently delayed” includes all young children whose scores on the MDI scales in infancy and toddlerhood never rose above − 1 SD. “Improving” includes all young children whose scores on the MDI scales fell below − 1 SD in infancy and then rose above − 1 SD in toddlerhood. “Deteriorating” includes all young children whose scores on the MDI scales were above − 1 SD in infancy and then fell below − 1 SD in toddlerhood. Control variables include the child’s age and gender, whether the child has siblings, whether the mother is the primary caregiver, whether the mother is more than 25 years old, whether the mother has attained 12 or more years of education, and the family asset index. We also control for baseline Bayley MDI scores, time and county fixed effect. Each column is a separate regression. Data source is authors’ survey

***p < .01

Table 5 presents the associations between developmental trajectories and preschool-age cognitive skills using Eq. (2), in which we regressed the persistently delayed, improving, and deteriorating trajectories on preschool-age FSIQ scores using the never delayed group as the reference group for comparison. Compared to children who never experienced cognitive delay, children with persistent cognitive delay and children with deteriorating cognitive development before age 3 had significantly lower preschool-age FSIQ scores by 0.73 SD and 0.52 SD, respectively. Children with improving cognitive development before age 3, however, demonstrated no significant differences in standardized FSIQ scores compared to children who were never delayed.

**Table 5:** Association between cognitive score at preschool age and trajectory of child cognitive development from infancy to toddlerhood (*N* = 1245)

Development	Standardized FSIQ scores (at preschool age)
Persistently delayed (1 = persistently, 0 = otherwise)	−0.73*** (0.11)
Improving (1 = improving, 0 = otherwise)	0.04 (0.13)
Deteriorating (1 = deteriorating, 0 = otherwise)	−0.52*** (0.06)
Control variables	Yes
County fixed effects	Yes
Time fixed effects	Yes
*R*-squared	0.25

Models estimated in this table are defined in Eq. (2) in the manuscript. “Never delayed” is the reference in the regression. “Never delayed” includes all young children whose scores on the Bayley Scale of Infant Development (*BSID*) of the Mental Development (*MDI*) in infancy and toddlerhood never fell below − 1 standard deviation (*SD*). “Persistently delayed” includes all young children whose scores on the MDI scales in infancy and toddlerhood never rose above − 1 SD. “Improving” includes all young children whose scores on the MDI scales fell below − 1 SD in infancy and then rose above − 1 SD in toddlerhood. “Deteriorating” includes all young children whose scores on the MDI scales were above − 1 SD in infancy and then fell below − 1 SD in toddlerhood. Control variables include the child’s age and gender, whether the child has siblings, whether the mother is the primary caregiver, whether the mother is more than 25 years old, whether the mother has attained 12 or more years of education, and the family asset index. We also control for baseline Bayley MDI scores, time, and county fixed effects. Data source is authors’ survey

***p < .01

### Associations between child and household characteristics and trajectory of child cognitive development

Table 6 presents the results of our multivariate probit analysis of the associations between child and household characteristics and cognitive trajectories before age 3, using Eq. (3). Columns 1 and 2 compare the characteristics of children who were never delayed versus those who had deteriorating cognitive development. The results show that children with older mothers, children whose mothers had higher education levels, and children from families with higher asset index scores were 9, 15 and 6% less likely to have deteriorating cognitive delay (rather than never having cognitive delay), respectively.

**Table 6:** Multivariate analysis of the association between characteristics and trajectory of child cognitive development from infancy to toddlerhood

Characteristic	Deteriorating		Improving	
	(1) β	(2) ME	(3) β	(4) ME
Child characteristics				
Age	− 0.02 (0.02)	−0.01 (0.01)	− 0.03 (0.05)	−0.01 (0.02)
Male (1 = yes)	0.08 (0.08)	0.03 (0.03)	0.01 (0.19)	0.00 (0.06)
Premature (1 = yes)	−0.06 (0.21)	−0.02 (0.08)	0.38 (0.35)	0.12 (0.11)
Have siblings (1 = yes)	0.02 (0.11)	0.01 (0.04)	0.35 (0.22)	0.11 (0.07)
Household characteristics				
Primary caregiver (1 = mother)	0.07 (0.09)	0.02 (0.03)	−0.34 (0.21)	−0.10 (0.07)
Maternal age (1 = more than 25 years old)	−0.25*** (0.09)	−0.09*** (0.03)	0.48** (0.22)	0.15** (0.07)
Maternal education level (1 = 12 years or higher)	−0.41*** (0.12)	−0.15*** (0.04)	0.75*** (0.25)	0.23*** (0.07)
Family asset index	−0.17*** (0.04)	−0.06*** (0.01)	0.32*** (0.09)	0.10*** (0.03)
County fixed effects	Yes	Yes	Yes	Yes
Time fixed effects	Yes	Yes	Yes	Yes
Observations	997	997	248	248

Models estimated in this table are defined in Eq. (3) in the manuscript. Column 1 presents coefficients and standard errors (in parentheses) from the probit regression. Column 2 presents marginal effects from the same probit regression, where 1 = “Deteriorating” and 0 = “Never” when child’s age is from 6 to 12 months (infancy) to 22 to 30 months (toddlerhood). The same multivariate analysis for “Improving” and “Persistent” are shown in Columns 3 and 4, where 1 = “Improving” and 0 = “Persistently delayed.” All regressions control for county fixed effects and time fixed effects. Data source is authors’ survey

**p < .05, ***p < .01

Columns 3 and 4 of Table 6 compare the child and household characteristics of children who were persistently delayed and those who showed improving cognitive development before age 3. The results find that the same characteristics—maternal age, maternal education level, and family asset index—were significantly positively correlated with improving cognitive development relative to children in the persistently delayed group. Children with older mothers, children more educated mothers, and children from families with higher asset index scores were 15, 23, and 10% more likely to have improving cognitive development rather than experiencing persistent cognitive delay, respectively. We found no statistically significant associations between child characteristics and any cognitive trajectory (Rows 1–4).

### Associations between cognitive scores at preschool age and in infancy and toddlerhood

Table 7 compares the associations of cognitive skills in infancy and toddlerhood, and cognitive trajectories before age 3, to preschool-age cognition using Eq. (4). The data show that the standardized cognitive scores in infancy and toddlerhood were significantly positively correlated with standardized FSIQ scores at preschool age. More precisely, a 1-SD rise in the standardized MDI score in infancy (6–12 months) was correlated with a 0.15-SD increase in standardized FSIQ scores at preschool age. When children were in toddlerhood (22–30 months), a 1-SD rise in the standardized MDI score was correlated with a 0.39-SD increase in standardized FSIQ scores at preschool age. A test of the difference between the two coefficients finds that they are significantly different. Finally, a 1-SD rise in standardized MDI scores from infancy to toddlerhood was correlated with a 0.13-SD increase in the standardized FSIQ scores at preschool age, indicating that improving the cognitive status from infancy to toddlerhood could lead to better cognitive development at preschool age.

**Table 7:** Associations between standardized cognitive scores at preschool age and in infancy and toddlerhood (*N* = 1245)

Variable	Standardized cognitive scores (at preschool age)		
	(1)	(2)	(3)
Standardized cognitive scores (in infancy)	0.15*** (0.03)		
Standardized cognitive scores (in toddlerhood)		0.39*** (0.03)	
Changes in standardized cognitive scores from infancy to toddlerhood			0.13*** (0.02)
Controls	Yes	Yes	Yes
County fixed effects	Yes	Yes	Yes
Time fixed effects	Yes	Yes	Yes
*R*-squared	0.16	0.26	0.16

Models estimated in this table are defined in Eq. (4) in the manuscript. Control variables include the child’s age and gender, whether the child has siblings, whether the mother is the primary caregiver, whether the mother is more than 25 years old, whether the mother of the child had attained more than 12 years of education, and family asset index. We also control for time and county fixed effects. All standard errors account for clustering at the village level. Data source is authors’ survey

***p < .01

Comparing the *R*-squared coefficients of the models that predict preschool-age cognition (Tables 4, 5, and 7) may provide insight into what measurement approaches might be best identifying the children at highest risk for long-term delayed development. The *R*-squared values associated with the four regressions in Table 4 are all between 0.19 and 0.22, whereas in Table 5 (including all of the trajectories together), the *R*-squared value is 0.25, suggesting that the full model would provide a slightly higher-quality prediction. In Table 7, the *R*-squared coefficient associated with the regression of toddler cognitive scores on preschool cognition (0.26; Column 2) is higher than the *R*-squared values for infant cognitive scores (0.16; Colum 1) and the change in cognitive scores (0.16; Column 3), suggesting that the predictive power of a 3-year-old’s cognitive score is superior to the other measures. There is little difference between the predictive power of the best-fitting model using trajectories (Table 5) and the best-fitting model using cognitive scores (Table 7). The best-fitting model for the trajectory had an *R*-squared value of 0.25 (Table 5, Row 7). The *R*-squared value of the best-fitting regression in Table 7 is 0.26 (Column 2, Row 7). Statistical tests of goodness of fit do not find any differences.

## Discussion

We studied the trajectories of child cognitive development before 3 years of age in rural Western China and examined how these paths affect predict cognitive skills at preschool age. We described the cognitive development outcomes of children when they were in infancy (6–12 months), toddlerhood (22–30 months), and preschool age (49–65 months) and identified children who were never delayed, persistently delayed, had improving cognition and had deteriorating cognition before age 3. The empirical analysis also examined the associations between trajectories of cognitive development before age 3 and cognitive development skills at preschool age and identified risk factors (child and household characteristics) associated with each trajectory of cognitive development before age 3.

The results demonstrate that the prevalence of cognitive delay among rural infants (20%), toddlers (55%), and preschoolers (45%) is significantly higher than what one would expect for children in a healthy population (15%) [47, 55]. These findings are consistent with a number of recent empirical studies in rural China [26, 31–34, 56]. According to these studies, 39 to 49% of infants and toddlers between 6 and 36 months are cognitively delayed, and 37 to 57% of children at preschool age are cognitively delayed. Hence, our results, using three observations for the same cohort, concur with the cross-sectional studies in the literature, indicating that the cognitive delay of children during the first 5 years of life is a common problem across rural China.

The data also revealed that a large share of children had deteriorating cognitive development before age 3. Whereas only 13% of children had persistent cognitive delay, 41% of the sample saw their cognitive skills deteriorate, meaning that they developed cognitive delay as they aged from infancy (6–12 months) to toddlerhood (22–30 months). In contrast, only 7% of the sample children saw their cognitive skills improve (recovered from cognitive delay between infancy and toddlerhood). These findings suggest that sample children in rural China who were cognitively delayed in infancy (20% of the original sample) were less likely to recover from cognitive delay by the time they reached toddlerhood. Moreover, over half of the children who were not cognitively delayed in infancy became delayed by the time they reached toddlerhood.

Perhaps most importantly, the analysis demonstrates that different trajectories of child cognitive development before age 3 predict different levels of cognitive skills at preschool age. Children who were never cognitively delayed and children with improving cognitive trajectories had significantly higher levels of cognitive skills when they reached preschool age, whereas children who were persistently delayed and those with deteriorating cognitive trajectories during the first 3 years had relatively lower levels of cognitive skills at preschool age. Although there has not been a lot of work in this specific area, the findings are in line with at least two previous international studies [19, 23], which found that children who exhibit cognitive delay in early life (at 9–24 months old) have a higher likelihood of being cognitively delayed later in life (at 4–5 years old). The finding that “never” delayed and “improving” children in the sample show similar levels of cognition at preschool age indicates that identifying and addressing cognitive delays before age three may reduce the overall prevalence of cognitive delays and promote healthy long-term development among children in rural China.

In addition, we were interested in which measure was most predictive of cognitive development at preschool age: a child’s cognitive trajectory before age 3, cognitive development at infancy (6–12 months), or cognitive development at toddlerhood (22–30 months). The results indicate that the cognitive trajectory before age 3 has similar predictive power to a child’s level of cognitive development at 3 years. Although no study has considered this issue specifically, other studies have shown that a child’s level of cognition at 3 years predicts cognitive skills when a child is 5 years old. Specifically, research [6, 16, 57] has shown that the predictive power, measured as *R*-squared (goodness of fit) of the equation, using 3-year-old cognitive development to predict 5-year-old development, ranged from 0.36 to 0.77. More importantly, the finding that the prediction of the cognitive trajectory before age 3 to cognitive development at preschool age is the same as that of cognitive development at age 3 suggests that it may not be worth spending valuable resources to monitor the trajectory of child cognitive development unless the monitoring is helpful in inducing investment in children that would arrest deterioration and overcome the persistence of cognitive delay to enable young children to improve their trajectory. In the case of rural China, however, where our study finds 41% of children have deteriorating cognitive development before age 3, monitoring developmental trajectories in early childhood may help to identify vulnerable children and provide timely intervention.

Finally, we identified a relatively small number of individual characteristics associated with the socioeconomic status of the caregiver that predict improving or deteriorating cognitive trajectories before age 3. Children who had older mothers, more educated mothers, and lived in households with high family asset indices were less likely to experience deteriorating trajectories of cognitive development and were more likely to experience improving trajectories. Such a finding is consistent with previous international research that has investigated factors associated with child cognitive development at a single point in time [58–62]. The research found that older mothers, more-educated mothers, and higher socioeconomic status of the household were positively associated with better child cognitive development. For example, a study conducted in Ecuador in the early 2000s, using a sample of 3000 children aged 36 to 72 months from poor families found that household wealth and maternal education were associated with higher cognitive scores [62].

This study makes three contributions to the literature. First, the strengths of this study include its population-based sampling technique, large sample size, and rigorous child development testing, all of which increase confidence in the validity of our findings. Second, this is the first study to investigate the trajectories of child cognitive development and the association between these trajectories and preschool-age development in rural China; it is also one of only a few studies to do so internationally. Finally, this study examined factors associated with the different trajectories of child cognitive development in rural China, and these findings may provide specific indicators to target the children who are more vulnerable to delayed cognitive development. This information also may help researchers and policymakers to improve the interventions aimed at reducing the prevalence of child cognitive delay in the early years of life.

We also acknowledge two limitations of this study. First, although we document changes in cognitive development from infancy to preschool age, the data were collected in three survey waves, separated by intervals of nearly 2 years. As a result, this analysis may underestimate the true share of children who were affected by cognitive delay through early childhood. Second, although the samples in this study were randomly selected from the Qinba Mountain area of China, we do not consider our results to be statistically representative of the entire country or other rural regions. Future studies should examine the changes in cognitive development of early childhood over shorter intervals to better understand the trajectories of cognitive development in both the short and long terms. Moreover, future studies should continue to expand on the current study by using surveys of a wider scope and sampling populations from other rural areas in China that this study did not explore.

## Conclusion

We studied the trajectories of child cognitive development before 3 years of age in rural Western China and examined how these paths predict cognitive skills at preschool age. Drawing on longitudinal data from 1245 children and their families in 11 rural counties in Western China, the results found that 20% of children were cognitively delayed in infancy (6–12 months), 55% were delayed in toddlerhood (22–30 months), and 45% were delayed at preschool age (49–65 months). About 41% of children had a deteriorating cognitive trajectory from infancy to toddlerhood, whereas only 7% had an improving trajectory. Compared to children who had never experienced cognitive delay, children with persistent cognitive delay and those with deteriorating development before age 3 had significantly lower cognitive scores at preschool age. Children with improving development before 3 showed no similar levels of cognition at preschool age as children who had never experienced cognitive delay. Children with older mothers, children whose mothers had higher education levels, and children from families with higher asset index scores were less likely to have deteriorating cognitive development and more likely to having improving cognitive development before age 3.

Findings of this paper have clear implications for both policymakers and researchers. Considering the high rates of child cognitive delay in the first 5 years of life in rural China and examining the evidence in this paper in regard to the trajectories of child cognitive development during 0 to 3 years of age in rural China, we recommend that China’s government act to help families to improve the cognitive development of their children at an early age, especially for rural families and those families with low SES. Programs should be established to help families measure levels of child cognitive development when children are young and provide immediate intervention for children with delays, with special consideration for vulnerable communities such as poor households in rural China.

## Supplementary Information

**Additional file 1: Appendix Table A1.** Comparisons of children completed the cognitive assessments and children not completed the cognitive assessments. **Appendix Table A2.** Ordinary Least Squares regression estimates of the association between demographic characteristics and trajectories of cognitive development from infancy to toddlerhood.

## Data Availability

The data analysed in this study are available from the corresponding author upon reasonable request.

## Abbreviations

SD: Standard deviations
GDP: Gross Domestic Product
RMB: Ren Min Bi
BSID: Bayley Scales of Infant Development
MDI: Mental Development Index
WPPSI-IV: Wechsler Preschool and Primary Scale of Intelligence-Fourth Edition
FSIQ: Full-Scale Intelligence Quotient
OLS: Ordinary Least Squares

## Footnotes

1 In China, nationally designated poverty counties are areas that have been recognized by the central government as low-income areas in greater need of government support. The threshold for poverty county status is an annual per capita income of less than 2300 RMB, or about 1 U.S. dollar per person per day (The State Council Leading Group Office of Poverty Alleviation and Development, 2012).

2 The household assets index was constructed using polychoric principal component analysis based on the following variables: tap water, toilet, water heater, washing machine, computer, Internet, refrigerator, air conditioning, motorcycle or electronic bicycle, and automobile.

## References

[1] Almond D, Currie J (2011). Human capital development before age five. Handbook of labor economics.

[2] Bornstein MH, Britto PR, Nonoyama-Tarumi Y, Ota Y, Petrovic O, Putnick DL (2012). Child development in developing countries: introduction and methods. Child Dev.

[3] Dong Q (2017). “Developing children’s cognitive capital, promoting social prosperity and progress” seminar, Beijing.

[4] Shonkoff J, Phillips D (2000). Setting the stage. From neurons to neighborhoods: the science of early childhood development.

[5] Young ME, Mustard F, Garcia M, Pence A, Evans JL (2008). Brain development and ECD: a case for investment. Africa’s future, Africa’s challenge: early childhood care and development in Sub-Saharan Africa.

[6] Fagan JF, Holland CR, Wheeler K (2007). The prediction, from infancy, of adult IQ and achievement. Intelligence.

[7] McCall RB, Carriger MS (1993). A meta-analysis of infant habituation and recognition memory performance as predictors of later IQ. Child Dev.

[8] Rose SA, Feldman JF (1995). Prediction of IQ and specific cognitive abilities at 11 years from infancy measures. Dev Psychol.

[9] Walker D, Greenwood C, Hart B, Carta J (1994). Prediction of school outcomes based on early language production and socioeconomic factors. Child Dev.

[10] Knudsen EI, Heckman JJ, Cameron JL, Shonkoff JP (2006). Economic, neurobiological, and behavioral perspectives on building America’s future workforce. Can J Behav Sci.

[11] Cunha F, Heckman J (2007). The technology of skill formation. Am Econ Rev.

[12] Heckman JJ (1995). Lessons from the bell curve. J Polit Econ.

[13] Mortensen EL, Andresen J, Kruuse E, Sanders SA, Reinisch JM (2003). IQ stability: the relation between child and young adult intelligence test scores in low-birthweight samples. Scand J Psychol.

[14] Schneider W, Wolke D, Schlagmüller M, Meyer R (2004). Pathsways to school achievement in very preterm and full term children. Eur J Psychol Educ.

[15] Rubin RA, Balow B (1979). Measures of infant development and socioeconomic status as predictors of later intelligence and school achievement. Dev Psychol.

[16] Rose SA, Feldman JF, Wallace IF, McCarton C (1991). Information processing at 1 year: relation to birth status and developmental outcome during the first 5 years. Dev Psychol.

[17] McCall RB (1984). Developmental changes in mental performance: the effect of the birth of a sibling. Child Dev.

[18] McCall RB, Appelbaum MI, Hogarty PS (1973). Developmental changes in mental performance. Monogr Soc Res Child Dev.

[19] Cheng ER, Palta M, Kotelchuck M, Poehlmann J, Witt WP (2014). Cognitive delay and behavior problems prior to school age. Pediatrics.

[20] Eisenhower AS, Baker BL, Blacher J (2009). Children’s delayed development and behavior problems: impact on mothers’ perceived physical health across early childhood. Soc Sci Med.

[21] Feinberg E, Silverstein M, Donahue S, Bliss R (2011). The impact of race on participation in part C early intervention services. J Dev Behav Pediatr.

[22] Halfon N, Houtrow A, Larson K, Newacheck PW (2012). The changing landscape of disability in childhood. Future Child.

[23] McManus BM, Rosenberg SA (2012). Does the persistence of development delay predict receipt of early intervention services?. Acad Pediatr.

[24] Witt WP, Gottlieb CA, Hampton J, Litzelman K (2009). The impact of childhood activity limitations on parental health, mental health, and workdays lost in the United States. Acad Pediatr.

[25] Lu C, Black MM, Richter LM (2016). Risk of poor development in young children in low-income and middle-income countries: an estimation and analysis at the global, regional, and country level. Lancet Glob Health.

[26] Wang L, Liang W, Zhang S, Jonsson L, Li M, Yu C, Sun Y, Ma Q, Bai Y, Abbey C, Luo R, Yue A, Rozelle S (2019). Are infant/toddler developmental delays a problem across rural China?. J Comp Econ.

[27] Xie Y, Zhou X (2014). Income inequality in today’s China. Proc Natl Acad Sci U S A.

[28] Gu Q, Gao M, Li Y, Wei X (2009). The survey search of the parenting behavior in migration workers (in Chinese). J Child Health Care.

[29] Xie S, Wang X, Yao Y (2006). The application of Bayley scales of infant development in infant nursing (in Chinese). J Nurs (China).

[30] Xu S, Huang H, Zhang J, Bian X (2011). Research on the applicability of Bayley scales of infant and toddler development-to assess the development of infants and toddlers in Shanghai (in Chinese). Chin J CHC.

[31] Wei QW, Zhang JX, Scherpbier RW, Zhao CX, Luo SS, Wang XL, Guo SF (2015). High prevalence of developmental delay among children under three years of age in poverty-stricken areas of China. Public Health.

[32] Yue A, Wang X, Yang S, Shi Y, Luo R, Zhang Q, Kenny K, Rozelle S (2017). The relationship between infant peer interactions and cognitive development: evidence from rural China. Chin J Sociol.

[33] Luo R, Yue A, Zhou H, Shi Y, Zhang L, Martorell R, Medina A, Rozelle S, Sylvia S (2017). The effect of a micronutrient powder home fortification program on anemia and cognitive outcomes among young children in rural China: a cluster randomized trial. BMC Public Health.

[34] Gan Y, Meng L, Xie J (2016). Comparison of school readiness between rural and urban Chinese preschool children. Soc Behav Pers.

[35] Luo R, Emmers D, Warrinnier N, Rozelle S, Sylvia S (2019). Using community health workers to deliver a scalable integrated parenting program in rural China: a cluster-randomized controlled trial. Soc Sci Med.

[36] Luo R, Zhang L, Liu C, Zhao Q, Deng M, Shi Y (2010). On the development of young children in Chinese poor rural areas. Stud Preschool Educ.

[37] Attanasio O, Cattan S, Fitzsimons E, Meghir C, Rubio-Codina M (2020). Estimating the production function for human capital: results from a randomized control trial in Colombia. SSRN Electron J.

[38] Rademeyer V, Jacklin L (2013). A study to evaluate the performance of black south African urban infants on the Bayley scales of infant development III. South Afr J Child Health.

[39] The State Council Leading Group Office of Poverty Alleviation and Development (2012). List of counties in contiguous poverty-stricken areas in China.

[40] National Bureau of Statistics of the People’s Republic of China (2017). National statistic yearbook 2017.

[41] Bayley N (1974). The Bayley scales of infant development: the manual scale.

[42] Hamadani JD, Baker-Henningham H, Tofail F, Mehrin F, Huda SN, Grantham-McGregor SM (2010). Validity and reliability of mothers’ reports of language development in 1-year-old children in a large-scale survey in Bangladesh. Food Nutr Bull.

[43] Nahar B, Hamadani JD, Ahmed T, Tofail F, Mehrin F, Huda SN (2009). Effects of psychosocial stimulation on growth and development of severely malnourished children in a nutrition unit in Bangladesh. Eur J Clin Nutr.

[44] Yi S, Luo X, Yang Z, Wan G (1993). The revising of the Bayley scales of infant development (BSID) in China. Chin J Clin Psychol.

[45] Bayley N (1969). Manual for the Bayley scales of infant development.

[46] Yi S (1995). Manual of Bayley scales of infant development, Chinese revision.

[47] Wechsler D (2012). Wechsler preschool and primary scale of intelligence-fourth edition.

[48] Li Y, Zhu J, Wechsler D (2014). Wechsler preschool and primary scale of intelligence-fourth edition (WPPSI-IV) Chinese version.

[49] Chen HY, Chen YH, Liao YK, Chen HP, Lynn R (2017). Dysgenic fertility for intelligence and education in Taiwan. Intelligence.

[50] Liu C, Lu L, Zhang L, Luo R, Sylvia S, Medina A, Rozelle S, Smith DS, Chen Y, Zhu T (2017). Effect of deworming on indices of health, cognition, and education among schoolchildren in rural China: a cluster-randomized controlled trial. Am J Trop Med Hyg.

[51] Lynn R (2009). What has caused the Flynn effect? Secular increases in the development quotients of infants. Intelligence.

[52] Trahan LH, Stuebing KK, Fletcher JM, Hiscock M (2014). The Flynn effect: a meta-analysis. Psychol Bull.

[53] Wang A, Zhou L, Zhang H (2016). The Flynn effect on intelligence test for children in China and its impacting factors. China Examinations.

[54] Rubio-Codina M, Araujo MC, Attanasio O, Muñoz P, Grantham-McGregor S (2016). Concurrent validity and feasibility of short tests currently used to measure early childhood development in large scale studies. PLoS One.

[55] Boyle CA, Decoufle P, Yeargin-Allsopp M (1994). Prevalence and health impact of developmental disabilities in US children. Pediatrics.

[56] Zhou T. Analysis of intelligence test results of 207 preschoolers in Zhangjiagang City (in Chinese): Chinese Community Doctor (Medical Specialty). 2009;05:120.

[57] Cohen SE, Parmelee AH (1983). Prediction of five-year Stanford-Binet scores in preterm infants. Child Dev.

[58] Ayoub C, O’Connor E, Rappolt-Schlictmann G, Vallotton C, Raikes H, Chazan-Cohen R (2009). Cognitive skill performance among young children living in poverty: risk, change, and the promotive effects of early head start. Early Child Res Q.

[59] Conger RD, Donnellan MB (2007). An interactionist perspective on the socioeconomic context of human development. Annu Rev Psychol.

[60] Eriksen HLF, Kesmodel US, Underbjerg M, Kilburn TR, Bertrand J, Mortensen EL (2013). Predictors of intelligence at the age of 5: family, pregnancy and birth characteristics, postnatal influences, and postnatal growth. PLoS One.

[61] Fergusson DM, Lynskey MT, Horwood LJ (1993). Conduct problems and attention deficit behaviour in middle childhood and cannabis use by age 15. Aust N Z J Psychiatry.

[62] Paxson C, Schady N (2007). Cognitive development among young children in Ecuador: the roles of wealth, health, and parenting. J Hum Resour.

