# Supplementary material for: Trajectories of child cognitive development during ages 0–3 in rural Western China: prevalence, risk factors and links to preschool-age cognition
Source: BMC Pediatr. 2021 Apr 26;21:199. doi: 10.1186/s12887-021-02650-y (PMC8074422; doi:10.1186/s12887-021-02650-y)
Supplement: Supplementary file 1 — Additional file 1: Appendix Table A1. Comparisons of children completed the cognitive assessments and children not completed the cognitive assessments. Appendix Table A2. Ordinary Least Squares regression estimates of the association between demographic characteristics and trajectories of cognitive development from infancy to toddlerhood. [file 12887_2021_2650_MOESM1_ESM.docx]

**Supplementary Appendix to**

**Trajectories of child cognitive development during ages 0–3 in rural Western China: Prevalence, risk factors and links to preschool-age cognition**

| **Appendix Table A1. Comparisons of children completed the cognitive assessments and children not completed the cognitive assessments** | | | | | | | | | |
| --- | --- | --- | --- | --- | --- | --- | --- | --- | --- |
|  | Baseline (Infancy) | | | Follow-Up 1 (Toddlerhood) | | | Follow-Up 2 (Preschool-Age) | | |
|  | Children not completed the cognitive assessments | Children completed the cognitive assessments | Diff.  (1)-(2) | Children not completed the cognitive assessments | Children completed the cognitive assessments | Diff.  (4)-(5) | Children not completed the cognitive assessments | Children completed the cognitive assessments | Diff.  (7)-(8) |
|  | Mean | Mean | P-value | Mean | Mean | P-value | Mean | Mean | P-value |
|  | (SD) | (SD) |  | (SD) | (SD) |  | (SD) | (SD) |  |
| Characteristic | (1) | (2) | (3) | (4) | (5) | (6) | (7) | (8) | (9) |
| Child characteristics |  |  |  |  |  |  |  |  |  |
| Age | 9.49 | 9.45 | 0.93 | 27.39 | 27.44 | 0.87 | 56.32 | 56.88 | 0.39 |
|  | (1.97) | (1.81) |  | (1.71) | (1.77) |  | (2.11) | (3.40) |  |
| Male | 0.52 | 0.51 | 0.96 | 0.52 | 0.51 | 0.96 | 0.52 | 0.51 | 0.96 |
| (1 = yes) | (0.51) | (0.50) |  | (0.51) | (0.50) |  | (0.51) | (0.50) |  |
| Premature | 0.00 | 0.05 | 0.25 | 0.00 | 0.05 | 0.25 | 0.00 | 0.05 | 0.25 |
| (1 = yes) | (0.00) | (0.21) |  | (0.00) | (0.21) |  | (0.00) | (0.21) |  |
| Have siblings | 0.19 | 0.24 | 0.51 | 0.19 | 0.24 | 0.51 | 0.19 | 0.24 | 0.51 |
| (1 = yes) | (0.40) | (0.43) |  | (0.40) | (0.43) |  | (0.40) | (0.43) |  |
| Household characteristics |  |  |  |  |  |  |  |  |  |
| Primary caregiver | 0.81 | 0.85 | 0.62 | 0.59 | 0.62 | 0.75 | 0.70 | 0.63 | 0.43 |
| (1 = mother) | (0.40) | (0.36) |  | (0.50) | (0.48) |  | (0.47) | (0.48) |  |
| Maternal age | 0.59 | 0.62 | 0.79 | 0.59 | 0.62 | 0.79 | 0.59 | 0.62 | 0.79 |
| (1 = more than 25 years old) | (0.50) | (0.49) |  | (0.50) | (0.49) |  | (0.50) | (0.49) |  |
| Maternal education level | 0.11 | 0.15 | 0.57 | 0.11 | 0.15 | 0.57 | 0.11 | 0.15 | 0.57 |
| (1 = 12 years or higher) | (0.32) | (0.36) |  | (0.32) | (0.36) |  | (0.32) | (0.36) |  |
| Family asset index | -0.37 | -0.05 | 0.16 | -0.37 | -0.05 | 0.16 | -0.65 | -0.33 | 0.16 |
|  | (1.32) | (1.17) |  | (1.32) | (1.17) |  | (1.44) | (1.19) |  |
| Observations | 27 | 1245 |  | 27 | 1245 |  | 27 | 1245 |  |
| *Notes*. Data source is author’s survey. | | | | | | | | | |

| **Appendix Table A2. Ordinary Least Squares regression estimates of the association between demographic characteristics and trajectories of cognitive development from infancy to toddlerhood.** | | |
| --- | --- | --- |
|  | Deteriorating | Improving |
| Characteristics | (1) | (2) |
| Child characteristics |  |  |
| Age | -0.01 | -0.00 |
|  | (0.01) | (0.01) |
| Male | 0.02 | -0.02 |
| (1 = yes) | (0.03) | (0.04) |
| Premature | 0.01 | 0.07 |
| (1 = yes) | (0.08) | (0.09) |
| Have siblings | -0.01 | 0.08 |
| (1 = yes) | (0.04) | (0.05) |
| Household characteristics |  |  |
| Primary caregiver | -0.01 | -0.12** |
| (1 = mother) | (0.04) | (0.05) |
| Maternal age | -0.07** | 0.13*** |
| (1 = more than 25 years old) | (0.04) | (0.05) |
| Maternal education level | -0.19*** | 0.26*** |
| (1 = 12 years of higher) | (0.05) | (0.06) |
| Family asset index | -0.08*** | 0.05** |
|  | (0.01) | (0.02) |
| County fixed effects | Yes | Yes |
| Time fixed effects | Yes | Yes |
| *R*-squared | 0.08 | 0.09 |
| Observations | 879 | 366 |
| *Note.* All trajectories are classified considering the Flynn effect. Column 1 presents coefficients and standard errors (in parentheses) from the OLS regression, where 1 = “Deteriorating” and 0 = “Never” when child’s age is from 6 to 12 months (infancy) to 22 to 30 months (toddlerhood). The same multivariate analysis for “Improving” and “Persistent” are shown in Columns2, where 1 = “Improving” and 0 = “Persistently delayed.” All regressions control for county fixed effects and time fixed effects.  ***p* < .05, ****p* < .01 | | |
